# Exogenous carbohydrate form during low-muscle glycogen conditions has minimal impact on cycling performance

**DOI:** 10.1080/15502783.2026.2629826

**Published:** 2026-02-26

**Authors:** Ian. R. Matthews, Alejandro M. Rosales, Josey K. Walker, Noah B. Wilfong, Rachel E. Perez, Brent C. Ruby, Dustin R. Slivka

**Affiliations:** a Montana Center for Work Physiology and Exercise Metabolism, School of Integrative Physiology and Athletic Training, The University of Montana, Missoula, MT, USA

**Keywords:** Energy gel, potato, pasta, fasting, sport nutrition, time trial

## Abstract

**Background:**

Skeletal muscle recovery is improved with immediate postexercise carbohydrate feeding. Little is known regarding muscle recovery and performance when feeding is delayed. The purpose of this study was to examine the effects of varying exogenous carbohydrates on cycling performance with delayed feedings and low skeletal muscle glycogen content.

**Methods:**

Following 60 min of cycling and an overnight fast (12.1 ± 0.4 h), the participants consumed 2.10 ± 0.13 g·kg^−1^ bodyweight carbohydrate of one of the following: whole potatoes (POT), cooked pasta (PAS), energy gel (GEL) or remained unfed (control, CON), then rested for 120 min. The participants then cycled for 60 min at 65% W_max_ and completed a 19.4 km time trial. Muscle and blood samples were collected prefeeding, 120 min postfeeding, and after cycling for glycogen, glucose, and insulin analyses.

**Results:**

The time trial mean power output was higher in the PAS (213 ± 56W, *p* = 0.006) and GEL (209 ± 71W, *p* = 0.011) compared to CON (179 ± 68W), but POT (196 ± 51W, *p* = 0.199) was not different from CON. Power was similar between POT, PAS, and GEL (*p* > 0.05). Time trial finish time trended towards significance (*p* = 0.088) with carbohydrate trials averaging 3 min faster than CON. Muscle glycogen was similar between trials (*p* = 0.446) and did not change due to feeding (prefeeding: 44 ± 21 mmol·kg^−1^, postfeeding: 47 ± 23 mmol·kg^−1^, *p *= 0.120). Glycogen declined after cycling for 60 min (26 ± 16 mmol·kg^−1^, *p *< 0.001) compared to pre-feeding and post-feeding samples. Glucose and insulin were elevated in carbohydrate trials over CON 0–30 min post-feeding (*p *< 0.05).

**Conclusions:**

Varied pre-exercise exogenous carbohydrate sources effectively improve cycling time trial performance in a glycogen compromised state.

## 1 Introduction

The availability of skeletal muscle and hepatic glycogen is critical for sustaining exercise capacity and performance [1–4]. A single intense exercise bout or several consecutive training sessions can reduce skeletal muscle glycogen stores, thereby limiting performance [1,5]. Additionally, liver glycogen is substantially reduced after an overnight fast, further challenging endogenous carbohydrate availability [6]. When exercise begins in a glycogen-depleted state due to prior exercise, insufficient recovery, or dietary restriction, pre-exercise carbohydrate intake is essential to optimize performance [7]. Current guidelines recommend consuming between 1 and 4 g·kg^−1^ of body weight of carbohydrate 2–3 h before exercise to help replenish endogenous carbohydrate stores and to enhance exogenous carbohydrate availability [8,9].

Various forms of carbohydrates including solids, liquids, and gels have been shown to improve exercise performance [2,10–15]. Athletes often choose between manufactured products, such as energy gels, and carbohydrate-rich whole foods such as pasta or potatoes [16]. Energy gels offer convenience and rapid digestibility, while pasta and potatoes are easily accessible and cost-effective sources of carbohydrate. However, these whole food sources contain additional nutrients, such as protein, fiber, and sodium, which can influence the metabolic response [17]. Additional nutrients included in whole foods will affect the caloric and water content even when matching carbohydrate amount, which may impact overall nutrient availability. For example, higher fiber may slow gastric emptying and digestion [18], while protein coingested with carbohydrate can alter the insulin response and potentially enhance glycogen synthesis [19,20].

Although carbohydrate ingestion is widely recognized as essential for exercise performance [2,8,10,11] and postexercise recovery [21–23], most research has focused on carbohydrate feeding strategies when exercise begins with adequate glycogen stores or on immediate recovery after exercise when glycogen resynthesis rates are at their highest. Consequently, limited evidence exists regarding the effects of different pre-exercise carbohydrate sources when starting exercise in a glycogen-compromised state, several hours removed from prior activity. Furthermore, few studies have directly compared whole food and manufactured carbohydrate sources under these conditions [24,25]. Therefore, the purpose of this study was to directly compare exercise performance following pre-exercise feedings of pasta, potato, and energy gel-based carbohydrates, as well as an unfed control, when endogenous carbohydrate stores are compromised.

## 2 Materials and methods

### 2.1 Participants

Participants were recruited from the surrounding university area. Participants were recreationally active (self-reported 180+ minutes of aerobic exercise per week) and were willing to cycle at high intensities. Participants also self-reported being free of pregnancy, free of orthopedic issues limiting cycling, and were not allergic to potatoes or gluten. The recruited heterogeneous participant cohort permits any findings to be generalizable to most exercise populations. Before participation, all participants provided written informed consent and completed the American College of Sports Medicine's preparticipation questionnaire. Before recruitment and testing, the University of Montana's Institutional Review Board approved all procedures (IRB# 162).

#### 2.1.1 Randomization protocol

Fourteen total individuals agreed to participate. One participant withdrew due to illness, and a second withdrew for personal health reasons. Thus, twelve individuals completed four experimental trials in a repeated measures crossover design at least one week apart. Participant identification numbers were randomly preassigned one of four counterbalanced trial sequences to prevent an order effect. This provided three participants in each of the four trial sequences. Randomization was automated in Excel (Microsoft, Redmond, WA, USA). A third participant was removed following study completion due to time trial amotivation. Eleven participants were subsequently used in analysis.

### 2.2 Preliminary testing

Participants arrived at the laboratory for preliminary testing after abstaining from food and caffeine for 2 h before their visit. Participants were not permitted to consume alcohol in the preceding 24 h. Nude body weight was determined using an electronic scale (PS-6600 ST, Befour Inc., Cedarburg, WI, USA), and standing height was measured unshod using a stadiometer (Hopkins Road Rod Portable Stadiometer, Hopkins Medical Products, Caledonia, MI, USA). Body composition was determined using bioelectric impedance after participants rested supine for 10 min (InBodyS10, InBody, Cerritos, CA, USA). Electrodes were placed on each ankle, as well as thumb and forefinger of each hand while participants remained still and quiet for the duration of the measurement (~120 s). Bioelectric impedance body composition analysis has an interclass correlation coefficient of ≥0.98 [26].

Peak oxygen uptake (VO_2peak_) and maximal power output (W_max_) were determined from a graded exercise test on an electronically braked cycle ergometer (Zwift Ride with Kickr Core 1, Wahoo Fitness, Atlanta, GA, USA). The protocol started at 95 Watts (W) for males and 60 W for females, increasing by 35 W every 3 min until volitional fatigue or a cadence dropping below 60 revolutions per min. Heart rate and expired gases were measured using a gas and flow calibrated metabolic cart (ParvoMedics, Inc., Salt Lake City, UT, USA). VO_2peak_ was calculated as the highest 30 s average oxygen uptake and W_max_ was calculated using the following equation: W_max_ = (Power output of final completed stage) + ((time in final stage before fatigue/total stage time) * 35), where power output is measured in W, time in final stage before fatigue is measured in seconds, total stage time is 180 s, and 35 is the difference in power (W) between each stage. The Kickr Core 1 smart trainer has a ±2% power accuracy [27].

### 2.3 Experimental design

The experimental trials were: 1) potatoes (Baby Bakers Roasted Potatoes, Simplot RoastWorks, Boise, ID, USA) (POT), 2) pasta (Rotini, Barilla, Parma, Italy) cooked in water and seasoned with garlic salt (Lawry's, McCormick & Co., Hunt Valley, MD, USA) and olive oil (Bertolli Extra Light Taste, Deoleo USA, Dallas, TX, USA) (PAS), 3) energy gel (Hammer Gel, Hammer Nutrition, Whitefish, MT, USA) (GEL), and 4) unfed control (CON). Five participants were provided with 2.25 g·kg^−1^ body weight carbohydrate, and six were given 2.0 g·kg^−1^ body weight carbohydrate based on individual ability to consume the volume of food in the allotted time. Sodium and fat content in PAS were matched to POT through the addition of garlic salt and olive oil. Nutrients were not added to the GEL condition. All food items were weighed uncooked on an electronic food scale (Taylor 3835, Taylor USA, Oak Brook, IL, USA) for accuracy with nutrition label serving sizes. Table 1 illustrates the detailed food items. Upon finishing their fourth trial, participants completed a food preference questionnaire where each trial was numerically ranked 1 through 4 from most preferred to least preferred. The ranking criteria was individually interpreted and did not provide resolution on specific components such as taste or gastrointestinal discomfort.

**Table 1.** Nutrients, energy, and weight of each feeding (potato, pasta, gel) and an unfed control. Data presented as mean ± SD.

	Control	Potato	Pasta	Gel
Carbohydrate (g)	0 ± 0	152 ± 32	152 ± 32	152 ± 32
Fat (g)	0 ± 0	15 ± 3	15 ± 3	0 ± 0
Protein (g)	0 ± 0	30 ± 6	25 ± 5	0 ± 0
Sodium (mg)	0 ± 0	1623 ± 347	1623 ± 347	181 ± 39
Total energy (kcal)	0 ± 0	913 ± 195	829 ± 177	652 ± 139
Prepared weight (g)	0 ± 0	850 ± 189	437 ± 97	244 ± 54

#### 2.3.1 Pre-trial ride

The evening prior to each experimental trial (start time 19:00–20:00 local time), participants cycled for 60 min at 65% of their W_max_, which was calculated from the graded exercise test on the preliminary testing day. Participants did not eat or consume caffeine in the 2 h prior to the visit. They also avoided strenuous exercise and alcohol consumption within 24 h of the pre-trial ride. This ride was designed to reduce skeletal muscle glycogen stores. Between completing the pre-trial ride and return to the laboratory for the experimental trial, participants fasted 12.1 ± 0.4 h. *Ad libitum* water consumption was permitted before returning to the lab the next morning.

#### 2.3.2 Experimental trial

The morning following the pre-trial ride, venous blood and muscle biopsy samples were immediately collected upon participant arrival (prefeeding), and one of the feedings was provided. When fed, participants were given 20 min to complete each feeding. During the CON (unfed) trial, participants sat quietly for 20 min to ensure the same time course between trials. A rest period of 120 min started immediately after the feeding period in all trials. Participants consumed 700 mL of room temperature water throughout the feeding and rest period. Four additional blood samples were collected at minutes 15, 30, 60, and 120 of the rest period. A second muscle biopsy sample was collected at 120 min. Ten minutes after the second muscle sample collection, participants began cycling at a constant work rate set to 65% of W_max_ for 60 min, corresponding to 73% ± 6% of maximal O_2_ uptake. For two participants, power output was adjusted in all trials during the constant work rate ride to ensure completion of the ride and match intensity between trials. Participants consumed an additional 700 mL of room temperature water during the constant work rate ride. Immediately after this ride, a final blood and muscle sample was taken (195 ± 3 min after feeding) and a 30 min rest period began. Participants then completed a 19.4 km time trial where they were instructed to complete the trial as quickly as possible and were able to self-select the resistance of the cycle ergometer. All rides for each trial were completed on the same cycle ergometer. The time trial was completed on a virtual course (Zwift, Long Beach, CA, USA). Each participant's height and weight were input into the software prior to each time trial. The virtual bicycle specifications were the Zwift time trial frame and 32 mm Carbon wheelset. Time to complete the time trial, mean power output, and mean heart rate were recorded at the completion of the time trial. Water consumption was not permitted during the time trial.

#### 2.3.3 Blood sampling

Six venipuncture blood samples were collected during each experimental trial from an antecubital vein. Blood was drawn prior to feeding, 15, 30, 60, and 120 min after feeding as well as directly after the constant work rate ride. A total of 6  mL of blood was collected into K2E K2EDTA tubes (Greiner Bio-One, Monroe, NC, USA) and inverted five times. The samples were immediately spun at 1300 × g for 10 min in a 4 °C refrigerated centrifuge (Jouan Inc., MR22i, Winchester, VA, USA). Four aliquots of plasma were prepared and stored at −30 °C until glucose and insulin analyzes.

#### 2.3.4 Muscle biopsies

Three muscle samples were collected from the *vastus lateralis* from the same incision site at three different angles (distal to proximal) to ensure sampling from different areas of the muscle during each experimental trial. The leg used for collection alternated each experimental trial. Samples were taken prefeeding, 120 min after feeding, and immediately after the constant work rate ride. Before the first and second biopsy, 3–5 mL of 1% lidocaine (without epinephrine) was injected subcutaneously and near the muscle fascia. Following anesthetization, an approximately 0.5 cm incision was made through the skin. A 14-gauge ProMag Ultra Biopsy Needle (Argon Medical Devices Inc., Athens, TX, USA) was inserted into the muscle belly to collect ~10–20 mg of muscle tissue. Upon extraction, the muscle sample was quickly frozen in liquid nitrogen and stored at −80 °C until glycogen analysis.

#### 2.3.5 Heart rate and gas collection

Heart rate was measured using a chest strap (graded exercise test) and upper arm strap (constant work rate ride, time trial) (Polar Electro, Kempele, Finland). Heart rate in the constant work rate ride and the time trial is expressed as an average of each ride. Measures of heart rate using both chest and upper arm straps have mean absolute percent error values of 1.35% [28]. VO_2_ and VCO_2_ were measured during the graded exercise test and from 25 to 30 min and 55 to 60 min during the constant work rate ride. Expired gases were used to calculate the respiratory exchange ratio (RER) and substrate use. Substrate oxidation rates were calculated using Péronnet and Massicotte's equation: Carbohydrate oxidation (g·min^−1^) = (4.585⋅VCO_2_) − (3.226 ⋅ VO_2_), Fat Oxidation (g·min^−1^) = (1.695 ⋅ VCO_2_) − (1.701 ⋅ VO_2_) [29]. Gas exchange data from the final 3 min of the two gas collection periods during the constant work rate ride are expressed as a single aggregate average.

### 2.4 Sample analyzes

Duplicate plasma samples were analyzed for insulin via enzyme-linked immunosorbent assay (ELISA). A commercial insulin ELISA kit (EIA-2953, DRG International Inc., Springfield, NJ, USA) was used according to the manufacturer's specifications. The microplates were automatically washed (1575 Immunowash Plate Washer, Bio-Rad, Hercules, CA, USA) and read at 450 nm with a 620 nm background subtraction (VersaMax Microplate Reader, Molecular Devices, San Jose, CA, USA). A standard curve was completed with each insulin microplate and plotted on a 4-paramater logistic regression. Duplicate plasma samples were analyzed for glucose via colorimetric assay. A commercial colorimetric glucose kit (MA-GLU-1, LifeSpan RayBiotech Life Inc., Peachtree Corners, GA, USA) was used following a 10-fold dilution (10 µL sample, 90 µL sample buffer) according to manufacturer's specifications. Glucose microplates were read at 500 nm with plate blank subtraction using the abovementioned microplate reader. A standard curve was completed for each glucose microplate and slope was determined using a linear regression fitting. All trials of plasma samples from any given participant were analyzed on the same 96-well microplate. The area under the curve was calculated for both insulin and glucose using the trapezoid method: ((data point_i_ + data point_i+1_)/2) * (sample time_-1_ − sample time_i_), where i represents a data point and corresponding sample time. The area under the curve was calculated in four segments (0–30, 30–60, 60–90, and 90–120 min). Two blood samples were collected during the 60–120 min period, and this area was divided in two for analysis. Insulin area under the curve values are expressed as pmol·L⁻¹·min and glucose AUC values are expressed as mmol·L⁻¹·min. Glucose and insulin analysis of prefeeding blood samples were used to confirm fasted values.

Muscle samples were analyzed for glycogen content according to manufacturer's specifications using an EnzyChrom™ Glycogen Assay Kit (BioAssay Systems, Heyward, CA, USA). Briefly, 12.9 ± 2.7 mg of muscle was homogenized in 500 µL of homogenization buffer (25 mM citrate, pH 4.2, 2.5 g·L^−1^ NaF) on ice in a 2 mL microcentrifuge tube using a handheld rotor stator style electronic homogenizer (Fisherbrand Homgenizer 150). Samples were further diluted (+250 mL) using the same homogenization buffer to ensure values were within the standard curve. Samples were then centrifuged at 14,000 × g for 5 min to remove debris and 10 µL added to 90 µL of kit working reagent on a 96-well clear bottom microplate in duplicate. The plate was allowed to incubate for 30 min at room temperature before being read on the abovementioned microplate plate reader at 570 nm. The values in µg·mL^−1^ were then converted to mmol·kg^−1^ wet tissue weight using the weight of the sample and an assumed molecular weight of glycogen glucosyl units of 162 g·mol^−1^.

### 2.5 Statistical analyzes

A one-way analysis of variance (ANOVA, Trial) with repeated measures was used to determine differences in time trial performance, gas exchange, and glucose and insulin AUC. Two-way ANOVAs with repeated measures (Trial × Time) were used to determine differences in glucose, insulin, and muscle glycogen content. In the event of a significant ANOVA, a Fisher's protected least significant difference post hoc analysis was completed to determine where significance occurred. Effect sizes were calculated using partial eta-squared (*ƞ*^2^_p_), with *ƞ*^2^_p_ = 0.01 designated as a small effect, *ƞ*^2^_p_ = 0.06 designated as a medium effect, and *ƞ*^2^_p_ = 0.14 as a large effect. A Freidman's test was used to detect differences in participant preference score. In the event of a significant Freidman's test, a Wilcoxon signed-rank test was used to determine where significant comparisons occurred. Statistical analysis was completed using the Statistical Package for Social Sciences (SPSS) (v. 29; IBM Corp., Armonk, NY, USA) with significance set at *p* < 0.05. Data are expressed as mean ± SD.

## 3 Results

### 3.1 Participant descriptives

Eight males and three females (*n* = 11) completed the study. Participants were 25 ± 6 years old, with a VO_2peak_ of 3.88 ± 1.07 L·min^−1^ and W_max_ of 310 ± 74 W. Descriptive data are presented in Table 2.

**Table 2.** Participant descriptive characteristics. Data are presented as mean ± SD. *n* = 11.

Participant characteristics	Mean ± SD
Age (years)	25 ± 6
Height (cm)	174.2 ± 11.7
Weight (kg)	73.4 ± 14.6
Body composition (% fat)	15.0 ± 10.2
VO_2peak_ (L·min^−1^)	3.88 ± 1.07
W_max_ (Watts)	310 ± 74

### 3.2 Constant work rate ride

**Figure 1.**
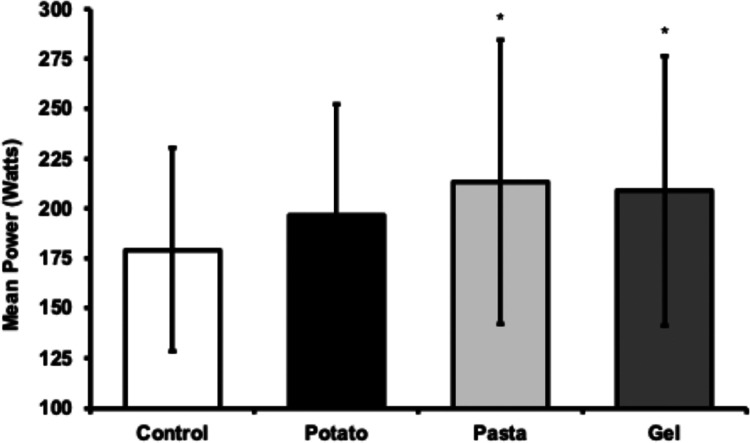
Mean power output throughout time trial in potato, pasta, gel trials, and an unfed control trial. *p < 0.05 compared to control. Data are presented as mean ± SD.

There were no differences in mean heart rate during the constant work rate ride between POT (156 ± 9 beats·min^−1^), PAS (156 ± 12 beats·min^−1^), GEL (156 ± 10 beats·min^−1^), and CON (160 ± 14 beats·min^−1^, *p* = 0.651, *ƞ*^2^_p_ = 0.052) trials. There were also no differences in VO_2_ between POT (2.73 ± 0.68 L·min^−1^), PAS (2.81 ± 0.64 L·min^−1^), GEL (2.78 ± 0.67 L·min^−1^), and CON (2.80 ± 0.72 L·min^−1^, *p* = 0.295, *ƞ*^2^_p_ = 0.114) trials. There was a large effect for RER (*p* < 0.001, *ƞ*^2^_p_ = 0.586), where RER was greater in POT (0.87 ± 0.02), PAS (0.87 ± 0.03), and GEL (0.87 ± 0.03) trials compared to the CON trial (0.83 ± 0.03, *p* < 0.001). RER was similar between POT, PAS, and GEL trials (*p* > 0.05). There was a large effect for carbohydrate oxidation between trials (*p* < 0.001, *ƞ*^2^_p_ = 0.522, Figure 1), with carbohydrate oxidized at a higher rate during POT (*p* < 0.001), PAS (*p* < 0.001), and GEL (*p* = 0.006) trials compared to the CON trial. Carbohydrate oxidation rate was similar between POT, PAS, and GEL trials (*p* > 0.05). Conversely, there was a large effect for fat oxidation between trials (*p* < 0.001, *ƞ*^2^_p_ = 0.504, Figure 1), with fat oxidation rates lower in POT (*p* < 0.001), PAS (*p* = 0.002), and GEL (*p* = 0.003) trials compared to the CON trial. Fat oxidation rate was similar between POT, PAS, and GEL trials (*p* > 0.05).

### 3.3 Time trial

**Figure 2.**
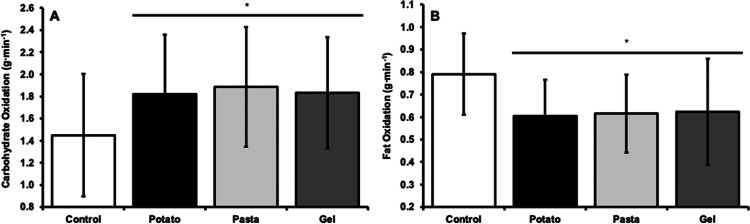
A. Carbohydrate and B. fat oxidation during the 60 min constant work rate ride in potato, pasta, gel trials, and an unfed control trial. Each bar represents an average of the 30 and 60 min collections. *p < 0.05 compared to control. Data presented as mean ± SD.

There was a large main effect for mean power output during the time trial (*p* = 0.010, *ƞ*^2^_p_ = 0.309, Figure 2A), where mean power output in PAS (*p* = 0.006) and GEL (*p* = 0.011) trials were higher compared to the CON trial. Mean power output in the POT trial was similar to CON (*p* = 0.199), PAS (*p* = 0.131), and GEL (*p* = 0.211) trials, and PAS and GEL trials were not different from each other (*p* = 0.666, Figure 2B). There was a large main effect for mean heart rate during the time trial (*p* = 0.024, *ƞ*^2^_p_ = 0.266), where PAS (163 ± 9 beats·min^−1^, *p* = 0.032) and GEL (160 ± 9 beats·min^−1^, *p* = 0.007) trials had a higher mean heart rate compared to the CON trial (153 ± 9 beats·min^−1^). Mean heart rate in the POT (159 ± 13 beats·min^−1^) trial was not different from CON (*p* = 0.101), and not different from PAS (*p* = 0.352), or GEL (*p* = 0.724) trials. Heart rate was not different between PAS and GEL trials (*p* = 0.376). There was no main effect for time to complete the trial, despite a trend towards significance (*p* = 0.088, *ƞ*^2^_p_ = 0.240). Time was faster on average in POT (33.3 ± 3.1 min), PAS (32.2 ± 3.2 min), and GEL (33.1 ± 4.9 min) trials compared to the CON (36.1 ± 8.0 min) trial.

### 3.4 Muscle glycogen

**Figure 3.**
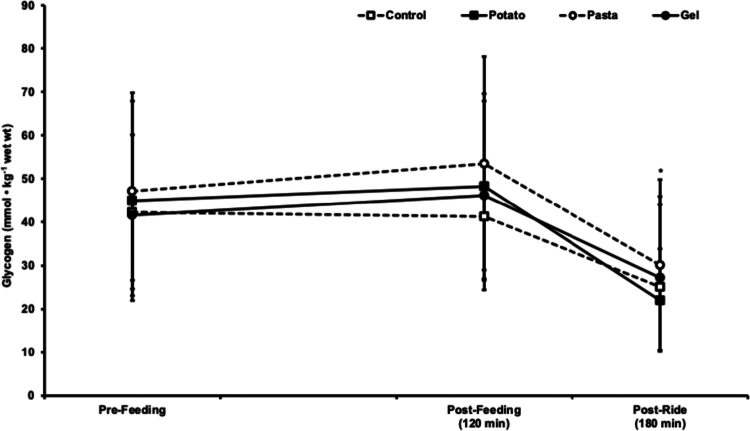
Muscle glycogen concentrations were collected prefeeding, postfeeding (120 min), and after a constant work rate (postride) ride in potato, pasta, and gel trials and an unfed control trial. *p < 0.05 Compared to pre-feeding and postfeeding. The data are presented as mean ± SD.

There were no differences in skeletal muscle glycogen concentration between trials (*p* = 0.446, *ƞ*^2^_p_ = 0.075). However, there was a large main effect for time (*p* < 0.001, *ƞ*^2^_p_ = 0.824). Skeletal muscle glycogen did not change from pre- to postfeeding in the initial 120 min (*p* = 0.120) but was lower after the constant work rate ride compared to both prefeeding and postfeeding samples (*p* < 0.001). Skeletal muscle glycogen values are presented in Figure 3.

### 3.5 Glucose

**Figure 4.**
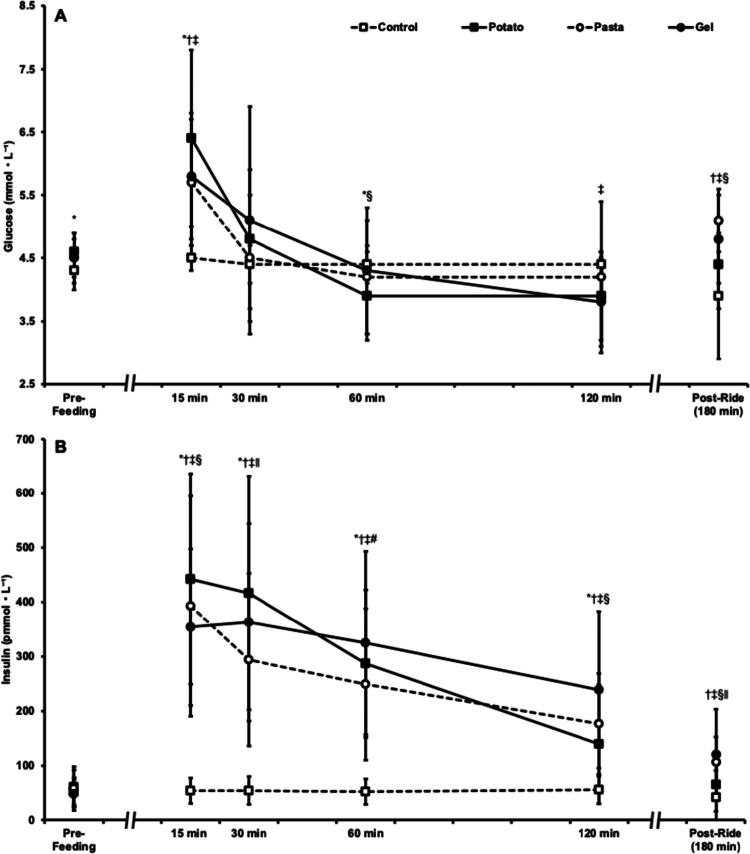
A. Plasma glucose and B. insulin throughout potato, pasta, and gel trials and an unfed control trial. *p < 0.05 compared to control in the potato trial, ^†^*p* < 0.05 compared to control in the pasta trial, ^‡^*p* < 0.05 compared to control in the gel trial, ^§^*p* < 0.05 compared to gel in the potato trial. ^∥^*p* < 0.05 compared to potato in the pasta trial. ^#^*p* < 0.05 compared to pasta in gel trial. Data are presented as the mean ± SD.

An interaction and large effect size was observed for plasma glucose (*p* < 0.001, η^2^_p_ = 0.374). The pre-feeding PAS, GEL, and CON trial plasma glucose levels were similar (*p* > 0.05); however, the POT trial was higher than the CON trial (*p* = 0.027). The pre-feeding POT, PAS, and GEL trial plasma glucose levels were similar (*p* > 0.05). At 15 min, plasma glucose in the POT (*p* < 0.001), PAS (*p* = 0.001), and GEL trials (*p* = 0.001) were higher than the CON trial; POT, PAS, and GEL were similar (*p* > 0.05). At 30 min, plasma glucose was similar between trials (*p* > 0.05). At 60 min, POT trial plasma glucose was lower than the CON trial (*p* = 0.032); PAS (*p* = 0.504) and GEL (*p* = 0.625) were not different than CON. At 60 min, POT trial plasma glucose was lower than the PAS trial (*p* = 0.019) but similar to the GEL trial (*p* = 0.217); PAS and GEL were similar (*p* = 0.914). At 120 min, POT (*p* = 0.074) and PAS (*p* = 0.683) trial plasma glucose were similar to the CON trial, but GEL was lower than CON (*p* = 0.042); POT, PAS, and GEL were similar (*p* > 0.05). Postride, POT trial plasma glucose was similar to the CON trial (*p* = 0.134), but the PAS (*p* = 0.006) and GEL trials (*p* = 0.009) were higher than the CON trial. Postride, POT trial plasma glucose level in the POT trial was lower than that in the PAS trial (*p* = 0.009) but similar to that in the GEL trial (*p* = 0.158); the PAS and GEL values were similar (*p* = 0.103). An interaction and large effect size were observed for the plasma glucose area under the curve (*p* < 0.001, η^2^_p_ = 0.507). The 0–30 min plasma glucose area under the curve in the POT (166 ± 29 mmol·L^−1^·min, *p* = 0.003), PAS (153 ± 20 mmol·L^−1^·min, *p* = 0.007), and GEL (160 ± 28 mmol·L^−1^·min, *p* = 0.010) trials were higher than those in the CON trial (133 ± 6 mmol·L^−1^·min); the POT, PAS, and GEL rates were similar (*p* > 0.05). The 30–60 min plasma glucose area under the curve was similar across all trials (POT, 131 ± 23; PAS, 130 ± 27; GEL, 140 ± 40; CON, 133 ± 8 mmol·L^−1^·min; *p* > 0.05). The 60–90 and 90–120 min plasma glucose areas under the curve in the POT trial (118 ± 19 mmol·L^−1^·min) were lower than those in the CON trial (132 ± 8 mmol·L^−1^·min, *p* = 0.026); the values of PAS (127 ± 30 mmol·L^−1^·min) and GEL (122 ± 22 mmol·L^−1^·min) were similar to those of the CON (*p* > 0.05). The 60–90 and 90–120 min plasma glucose areas under the curve were similar for the PAS and GEL (*p* = 0.509). The blood glucose data are presented in Figure 4A.

### 3.6 Insulin

An interaction with a large effect size was observed for plasma insulin (*p* < 0.001, η^2^_p_ = 0.628). Prefeeding plasma insulin was similar between trials (*p* > 0.05). At 15 min, POT, PAS, and GEL trial plasma insulin levels were higher than those in the CON trial (*p* < 0.001); POT and PAS were similar (*p* = 0.089), but POT was higher than GEL (*p* = 0.011). The results of the PAS and GEL trials were similar at 15  min (*p* = 0.403). At 30 min, POT, PAS, and GEL trial plasma insulin levels were higher than those in the CON trial (*p* < 0.001); POT was higher than PAS (*p* = 0.035), but POT and GEL were similar (*p* = 0.101). The results of the PAS and GEL trials were similar at 30 min (*p* = 0.140). At 60 min, POT, PAS, and GEL trial plasma insulin levels were higher than those in the CON trial (*p* < 0.001); POT was similar to PAS and GEL (*p* > 0.05). The PAS trial was lower than the GEL trial at 60  min (*p* = 0.017). At 120 min, POT (*p* = 0.020), PAS (*p* < 0.001), and GEL (*p* < 0.001) trial plasma insulin levels were higher than those in the CON trial; POT was similar to PAS (*p* = 0.094), but POT was lower than GEL (*p* = 0.002). The results of the PAS and GEL trials were similar at 120  min (*p* = 0.052). Post-ride, POT trial plasma insulin was similar to the CON trial (*p* = 0.212), but PAS (*p* = 0.020) and GEL (*p* = 0.016) were higher than CON. Post-ride, the POT trials were lower than the PAS (*p* = 0.025) and GEL (*p* = 0.033) trials; PAS and GEL were similar (*p* = 0.586). An interaction with a large effect size was observed for the plasma insulin area under the curve (*p* < 0.001, η^2^_p_ = 0.548). The 0–30 min plasma insulin area under the curve in the POT (10,144 ± 4,466 pmol·L^−1^·min), PAS (8,535 ± 4,145 pmol·L^−1^·min), and GEL trials (8,391 ± 3,472 pmol·L^−1^·min) was greater than that in the CON trial (1,674 ± 785 pmol·L^−1^·min, *p* < 0.001). The POT trial 0-30 min area under the curve was higher than the PAS (*p* = 0.031) and GEL trials (*p* = 0.007); the PAS and GEL values were similar (*p* = 0.854). The 30–60 min plasma insulin area under the curve was higher in the POT (10,553 ± 5085 pmol·L^−1^·min), PAS (8153 ± 4171 pmol·L^−1^·min), and GEL trials (10,322 ± 5038 pmol·L^−1^·min) than in the CON trial (1594 ± 706 pmol·L^−1^·min, *p* < 0.001). The POT trial 30–60 min plasma insulin area under the curve was higher than the PAS trial (*p* = 0.021) but similar to the GEL trial (*p* = 0.738); PAS was lower than GEL (*p* = 0.016). The 60–90 and 90–120 min plasma insulin areas under the curve in the POT (6388 ± 3487 pmol·L^−1^·min), PAS (6383 ± 3322 pmol·L^−1^·min), and GEL trials (8457 ± 4395 pmol·L^−1^·min) were higher than the CON trial (1612 ± 717 pmol·L^−1^·min, *p* < 0.001). The 60–90 and 90–120 min plasma insulin area under the curve in the POT trial was similar to the PAS trial (*p* = 0.991) but lower than the GEL trial (*p* = 0.008); PAS was lower than GEL (*p* = 0.003). The plasma insulin values are displayed in Figure 4B.

### 3.7 Food preference

Participant food preference rankings were different between trials, (χ^2^(3) = 12.709, *p* = 0.005) with the PAS feeding ranked highest (mean rank: 1.4 ± 0.7), followed by GEL (2.5 ± 1.0, *p* = 0.036 from PAS), POT (2.9 ± 1.0, *p* = 0.007 from PAS), and CON (3.2 ± 0.9, *p* = 0.006 from PAS). There were no differences in preference ranking between POT, GEL, and CON (*p* > 0.05).

## 4 Discussion

The purpose of the present work was to examine how various exogenous carbohydrate sources impact carbohydrate metabolism and cycling performance when prefeeding occurs removed from exercise and with reduced glycogen. Rationale for this work is justified by evidence that endurance exercise training and performance rely on a finite endogenous as well as a supply of exogenous carbohydrate sources [1–4,30,31]. As supported here [24,25,32,33], the exact exogenous carbohydrate source to support endurance exercise can be diverse, spanning whole foods to commercially marketed sport supplements. Efficacy was determined from common whole-food sources (potatoes, pasta), a commercially available energy gel, and an unfed control. The present results demonstrate that despite subtle differences in insulin and glucose dynamics due to feeding, varied pre-exercise carbohydrate sources benefit cycling time-trial performance. Interestingly, skeletal muscle glycogen content was not meaningfully resynthesized in the two hours following carbohydrate consumption in these conditions, signifying that blood glucose availability primarily supported time trial performance. Moreover, a whole food source was preferred by the participants. Thus, the period prior to exercise is a valuable window to ensure sufficient carbohydrate availability to support performance [34] under glycogen-compromised conditions, and the exact source consumed during this window can vary.

Depleted or limited skeletal muscle glycogen storage compromises aerobic exercise capacity and performance [1,2] and to thoroughly test each carbohydrate source, a pretrial ride and overnight fast were implemented to reduce the skeletal muscle glycogen content. This approach has practical importance by mimicking the consequences of serial glycogen depletion from training bouts or races without glycogen repletion from immediate postexercise carbohydrate feedings [5]. The novelty in this work thus lies in the examination of varied carbohydrate sources when prefeeding is removed from preceding exercise and performance trials begin with a reduced glycogen content. More replete levels of initial glycogen content could otherwise mask the performance impact of exogenous carbohydrate sources [35]. Initial skeletal muscle glycogen content in the current investigation was low due to exercise and fasting (grand mean: 44 ± 21 mmol·kg^−1^ wet weight) relative to normative resting values around 100 mmol·kg^−1^ wet weight [36].

Sport nutrition products are strategically formulated to deliver carbohydrates in small volumes to maximize absorption and avoid gastrointestinal distress [37]. Whole food options containing carbohydrates include other nutrients which may influence gastrointestinal response [17]. The food options selected here were expressly matched for carbohydrate and delivered in accordance with pre-exercise recommendations (1–4 g·kg^−1^ body weight) [9] but varied in caloric content and volume due to additional macronutrients and water content (Table 1). The potato and pasta trials were closely matched for fat, protein, and sodium, but additional nutrients were not added to the energy gel. Fewer total calories were delivered with the energy gel (652 ± 139 kcals) compared to the potato (913 ± 195 kcals) and pasta (829 ± 177 kcals) trials. The peak blood glucose, based on area under the curve (0–30 min), was similar among all carbohydrate feeding trials with insulin responding accordingly before decreasing. Notably, the peak insulin area under the curve profile (0–30 min) of the potato trial was elevated above the pasta and gel trials. This peak insulin in the potato trial transiently lowered blood glucose area under the curve below the control trial in the hour (60–120 min) before the constant work rate ride, signifying an incidence of reactive hypoglycemia not evident with the other carbohydrate trials. Protein and carbohydrate can together incite additive insulinemic responses compared to carbohydrate alone [19,20]. Despite matching the potato and pasta trials for protein content to account for insulinemic discrepancies, the volume of the potato trial may have slowed gastric emptying to prolong insulin elevation.

Insulin and exercise can stimulate glucose transporter 4 (GLUT4) translocation to the sarcolemma to facilitate glucose entry into skeletal muscle cells for eventual glycogen resynthesis [38–40]. Glycogen-depleting exercise combined with carbohydrate feedings provide potent stimuli for resynthesis of skeletal muscle glycogen [35,41]. Delaying feedings by >2 h post-exercise impairs resynthesis rates by up to 45% (23) due to GLUT4 relocating intracellularly towards preexercise levels [42], lowering glucose transport into muscle cells. In conjunction with the pretrial ride, participants fasted for ~15 h, well beyond common carbohydrate refeeding timing recommendations [36]. Although carbohydrate feedings caused transient rises in glucose and insulin during the 2-h rest/digestion period, the skeletal muscle glycogen content only marginally increased (14% ± 24%), and the increase was not different than the unfed control (3% ± 23%). Previous reports demonstrating skeletal muscle glycogen resynthesis do so with immediate postexercise feed (one to two) and oftentimes utilize a 4-h rest period [23,32,33,43]. The increase and decrease in plasma glucose without meaningful changes in skeletal muscle glycogen content suggests that blood glucose may have been stored hepatically.

Exercise aside, hepatic glycogen content is substantially reduced after an overnight fast [6] and hepatic uptake can account for a majority of ingested glucose postprandially [44] before being released into circulation. Although prefeeding blood glucose was elevated in the POT trial Compared to CON, pre-feeding blood glucose levels neared the lower overnight fasting normative range [45] at ≤4.5 mmol·L^−1^ in all trials. Peak blood glucose at 15 min was between 5.7 and 6.4 mmol·L^−1^ after 152 ± 32 g of carbohydrate, which are values expected with half the glucose load (75 g) of an oral glucose tolerance test [46]. Blood glucose values returning towards prefeeding levels after 30 min points towards hepatic storage precedence as opposed to skeletal muscle glycogen resynthesis. Hepatic glycogen appeared available for exercise (after release to the blood stream and subsequent uptake by the muscle) alongside any remaining skeletal muscle glycogen content. Carbohydrate oxidation rates were higher and fat oxidation rates were lower when participants were fed compared to unfed during the constant work rate ride and this likely carried over into the time trial to support performance.

Empirical works exhibiting improved exercise performance with pre-exercise carbohydrate intake commonly attribute these findings to skeletal muscle glycogen resynthesis and subsequent carbohydrate availability [11,15,47]. Since glycogen was not meaningfully synthesized here, irrespective of carbohydrate feeding, the time trial performance improvements are anticipated to have been mediated by blood glucose availability. This has similarly been demonstrated when carbohydrate is consumed concurrently during exercise as opposed to pre-exercise [25]. Energy gel or potato purée consumption during prolonged cycling can equivalently elevate blood glucose and carbohydrate oxidation compared to no food, and both sources can serve as a viable fuel to improve cycling time trial performance [25]. However, in this past study the potato purée consumed during exercise was processed and packaged to resemble a sports gel which could have improved absorption [25] as opposed to the whole potatoes consumed before exercise here. In the present work, blood glucose was higher following the constant work rate ride in the carbohydrate trials than the unfed control. Due to blood glucose availability, time trial mean power output was higher, with finish times improved by nearly 3 min on average. As a result, the potato, pasta, and energy gel time trials were generally completed at a higher heart rate than the unfed control. Thus, when skeletal muscle glycogen content is compromised, blood borne fuel availability is essential to support aerobic performance.

The importance of blood borne fuel remains evident when reviewing time trial outcomes in the potato and control trials in relation to blood glucose surrounding the constant work rate ride. Potato trial blood glucose area under the curve was smaller than the control trial in the hour (60–120 min) before the constant work rate ride, a comparative hypoglycemia to begin exercise. Blood glucose did numerically rebound following the constant work rate ride in the potato compared to the control trial likely due to hepatic output. Blood glucose was thereby an available fuel for the time trial in the potato trial but did not reach statistical significance compared to the control trial (*p* = 0.880). Accordingly, time trial mean power output (*p* = 0.199) and heart rate (*p* = 0.101) were numerically higher in the potato trial than the control trial but also did not reach significance. As both mean power output (η^2^_p_ = 0.309) and heart rate (η^2^_p_ = 0.498) exhibited large effect sizes, the importance of carbohydrate feedings that can adequately elevate blood glucose in a glycogen-compromised state was substantiated.

Following the completion of the study, the participants ranked each trial based on overall preference. Pasta was the most preferred trial, followed in order by gel, potato, and control. The ensuing rankings may be related to combination of individual presentation, taste, and/or gastrointestinal discomfort [48]. Pasta was consumed conventionally from a bowl and seasoned with olive oil and garlic salt to match the preseasoned sodium and fat content of the potatoes. Seasonings were not added with the intention to enhance taste or flavor. The energy gel was the smallest in total volume but was unconventionally consumed as a single bolus from a bowl with a spoon as opposed to the common ~25 g packets. Potatoes were preseasoned with garlic, salt, and olive oil, and consumed from a bowl. The potato trials had the highest prepared weight of the three feedings (Table 1) and caused gastric distress for some participants who remained throughout cycling. For this reason, the carbohydrate content of all the feedings was reduced from 2.25 to 2.0 g·kg^−1^ body weight for six of the eleven participants for all their trials. Regardless, the pasta preference together with the overall improvement in performance across carbohydrate sources indicates that whole foods can be considered and even preferred alongside commercially marketed sports meals peri-exercise.

The novelty in this work was the examination of varied carbohydrate sources when prefeeding begins with a reduced glycogen content. With respect to the parameters of reduced skeletal muscle glycogen and a prolonged fast, carbohydrate intake 2 h prior to a time trial improves performance when Compared to an unfed control. The mechanism producing these improvements appeared to be blood glucose availability as opposed to glycogen availability. The present results contribute to a growing body of literature displaying similar efficacy of whole foods compared to sport nutrition products before [14,24], during [25], and after [32,33] exercise. The present results suggest that carbohydrate intake is effective regardless of the consumption form, even when skeletal muscle glycogen is compromised. Therefore, athletes selecting pre-exercise carbohydrate-based meals can select foods based on carbohydrate content as opposed to specifically engineered products.
